# QCL Active Area Modeling with a View to Being Applied to Chemical Substance Detection Systems

**DOI:** 10.3390/s23010389

**Published:** 2022-12-30

**Authors:** Mariusz Mączka, Grzegorz Hałdaś, Stanisław Pawłowski

**Affiliations:** 1 Department of Electronics Fundamentals, Faculty of Electrical and Computer Engineering, Rzeszow University of Technology, 35-959 Rzeszow, Poland; 2 Department of Electrodynamics and Electrical Machine Systems, Faculty of Electrical and Computer Engineering, Rzeszow University of Technology, 35-959 Rzeszow, Poland

**Keywords:** Quantum cascade laser, chemical substance detection systems, non-equilibrium Green function (NEGF), QCL simulation

## Abstract

Numerical research into the QCL tunability aspects in respect to being applied in chemical substance detection systems is covered in this paper. The QCL tuning opportunities by varying power supply conditions and geometric dimensions of the active area have been considered. Two models for superlattice finite (FSML) and infinite (RSM) size were assumed for simulations. The results obtained have been correlated with the absorption map for selected chemical substances in order to identify the potential detection possibilities.

## 1. Introduction

**Figure 1:**
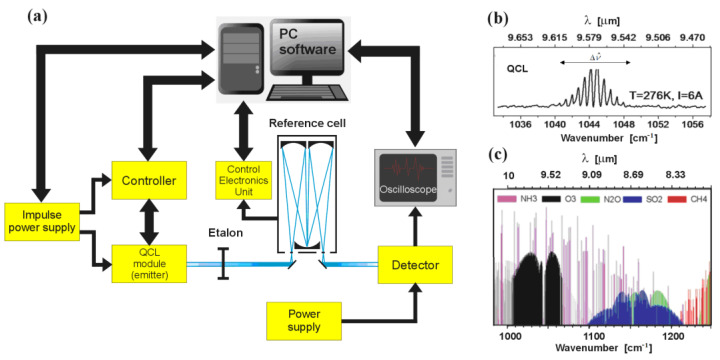
Illustration of the chemical detection method: (**a**) block diagram of a typical detection system with QCL module as emitter; (**b**) energy spectrum of the emitted laser beam for typical power supply conditions; (**c**) absorption spectrum of selected chemical compounds plotted on the basis of HITRAN.

Quantum cascade lasers (QCLs) are currently one of the most commonly used sources of IR radiation. They can be found in spectrometers [1,2,3,4], electronic countermeasures [5,6], telecommunication systems [7,8,9], or detectors of chemical substances [10,11,12,13,14]. The latter application seems to be greatly beneficial, as the combination of the detection method’s effectiveness and easily tunable QCL emitters provides detection systems that are both highly sensitive and selective. A typical example of such a system is shown in Figure 1, where the QCL module emitting a beam of light of a wavelength of λ ≈ 9.5 μm and a spectral width $\Delta \hat{\nu }\approx 10{\;[\mathrm{cm}}^{-1}]$ plays the main role (see Figure 1b). The range of this radiation covers absorption spectra of several chemical compounds such as NH_3_ or O_3_, as shown in the graph in Figure 1c.

The applied spectral analysis method determines the operation of the QCL module shown in Figure 1a. It can operate under an interpulse or an intrapulse tuning regime. Interpulse spectroscopy uses room-temperature lasers that generate ultra-short pulses of quasi-monochromatic radiation. Between the successive pulses of radiation, a change in the wavelength of the generated radiation in the selected spectral range occurs. Impulse operation, however, causes the radiation wavelength to undergo undesirable changes within the pulse duration, which results in the broadened spectral line of the device; hence, its spectral resolution is reduced. In order to minimise such phenomena, it is necessary to limit the width of the pulses to a maximum of tens of nm and to maintain their amplitude close to the laser excitation threshold. Thus, the QCL can be tuned in the range of 1–2 cm^−1^ with the pulse repetition period ranging from several dozen to several thousand hertz [15].

Intrapulse spectroscopy, similar to interpulse spectroscopy, is based on the analysis of the signal emitted by a pulsed laser at room temperature. Unlike the method described previously, changes in the frequency of the emitted wave during the pulse duration are not prevented, but upon determining their range, the tuning process is controlled by powering the laser at a level of several amperes above the excitation threshold. In such a case, the pulse width reaches several μm and the tuning range reaches 4–6 cm^−1^ at the pulse repetition frequency of up to 100 kHz [16].

Regardless of the spectral analysis method applied, it is the QCL tunability that determines how many and what types of substances are detected. Apart from the advantages of cascade lasers in terms of the emitted waves’ bandwidth (widely recognised by scientists [17,18,19] and emphasized by commercial producers as well), minor changes in the structure may significantly improve QCL parameters in this area. In particular, changes in the laser active area, whose spatial configuration has the most significant impact on the excitation conditions of the photon emission and its optical gain, may prove greatly beneficial. Computer simulations are the simplest and cheapest research tools that effectively help to test various QCL design variants and their impact on the most important optical parameters. Tunability of selected QCL structures has been tested under the study; the research has been aimed at assessing their potential application into gas detection systems.

By varying laser power supply conditions and the geometric dimensions of the laser active area, the laser tuning capabilities have been studied. The obtained results have been correlated with the absorption map for the selected chemical substances in order to assess whether their detection by the tested QCL module is feasible.

## 2. Numerical Models of QCL

**Figure 2:**
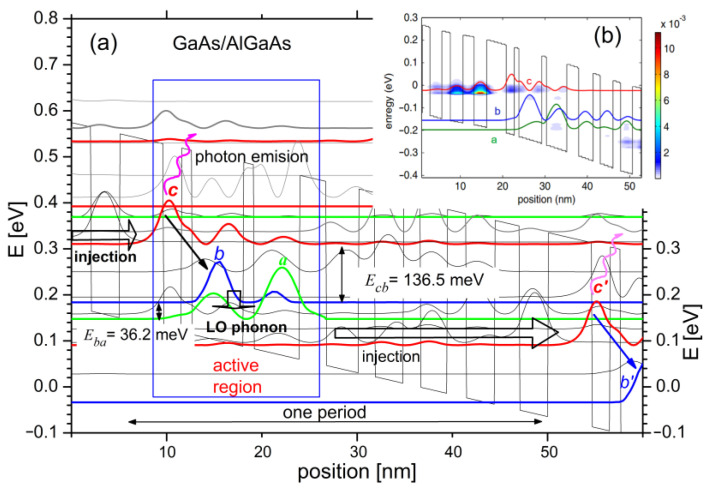
QCL operation presented: (**a**) basic electron transitions within the polarised laser structure calculated with FMSL model; (**b**) electron concentration distribution for a single QCL period determined with RSM model.

Two models assuming finite (FSML) and infinite (RSM) sizes of semiconductor superlattices were used to perform numerical studies. FSML, due to highly efficient simulations [20,21,22], was applied to carry out approximate calculations related to the device tuning capacity within a wide range of control voltage. The RSM [23,24] model was used to verify the research at the most important points, and all the vital electron scatterings were taken into account. The results were subjected to analysis, including transport and optical parameters calculated for the implemented laser [25] capable of operating at temperatures of about 300 K. Exemplary results are shown in Figure 2. Part (a) depicts a fragment of a voltage-polarized semiconductor superlattice represented by the energy distribution of the bottom conduction band E_c_, for which the energy states calculated with the FMSL model, significant for the quantum effects analysed in this paper, are plotted.

In the active region of the structure model, three quantum states were distinguished, namely, high—*c*, medium—*b* and low—*a* states. When the laser is under operation, the electrons injected from the previous period of the structure into the state *c* tend to transfer to the intermediate state *b* by emitting photons with energy equal to the energy difference between the subbands *c* and *b*. However, such transitions occur only for the population inversion, where the concentration of electrons in the level *c* exceeds their concentration in the miniband *b*. This can be confirmed by analysing the energy map of the electron charge concentration shown in Figure 2b, obtained with the RSM model.

The presented map shows a significant concentration of electrons at the *c* level in the laser active region, and a nearly invisible charge near the *b* state in the same region. This opens electronic photon transition opportunities that, after compensation for the waveguide losses, lead to the laser beam emission. This emission occurs above the J_TH_ threshold, which may be the beginning of the QCL tuning process within the chemical detection system using one of the detection methods described in the previous section.

**Figure 3:**
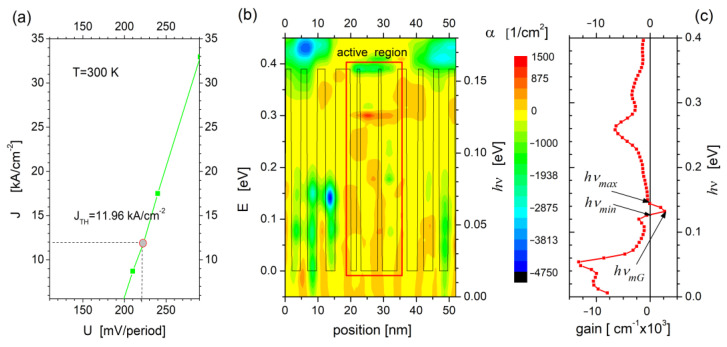
Simulation results of the QCL optical parameters calculated with RSM for the data in Table 1: Current–voltage characteristics and laser threshold current (**a**); the optical gain energy map (**b**) dependence of optical gain on the energy *hν* (**c**) for voltage U = 220 mV/period.

The value of the threshold current density (J_TH_ =11.95 kA/cm^2^) together with the current–voltage characteristics of the tested QCL structure presented in Figure 3a were determined with the RSM model for the parameters given in Table 1. The calculations were carried out with electron scattering due to crystal lattice disorder (AD), interface roughness (IR), scattering on impurity ions (ID), acoustic and optical phonons (AP and OP, respectively), as well as electron–photon (E–P) and electron–electron (E–E) interactions taken into account. Methods for incorporating electron scattering into calculations are described elsewhere [26,27,28,29].

Electron transitions between states *c* and *b* can be quantified by determining the optical gain of the laser under specific power conditions. An exemplary simulation for this parameter at the voltage of U = 210 mV/period is shown in Figure 3b. The energy map of the optical gain (*α* [cm^−2^]) plotted against the background of the potential representing the superlattice structure shows that the positive values of this parameter, characteristic for photon transitions, are essentially limited to the laser active region. In addition, the graph in Figure 3c shows the *hν* values for positive optical gain to be in the range of 126–139 meV, which corresponds to the calculations presented in Figure 1a. The described simulations, however, require a relatively large amount of time and computer power. Therefore, in order to quickly and effectively assess the range of emitted energy at *c* → *b* transitions, the FMSL model was applied to track the positions of the concerned states into the energy domain for changing supply voltage. The results are described in the next chapter.

Influenced by the electric field, the electrons in the laser active region are transmitted from the medium state *b* to the low state *a*, and their energy is transferred to the crystal lattice as phonons (see Figure 2a). The injection area of the next period of QCL is designed to transfer electrons from the active region low state *a* and ensure their further transport to the high state *c’* of the next QCL module, where the whole sequence of transitions between states is repeated (see Figure 2a *c*’→ *b*’). Such a QCL operation scheme turns each electron participating in the *E_cb_* transition into a multi-photons source, which results in relatively high output powers and is, along with a significant tunability, one of the greatest advantages of such devices.

## 3. QCL Tuning

**Figure 4:**
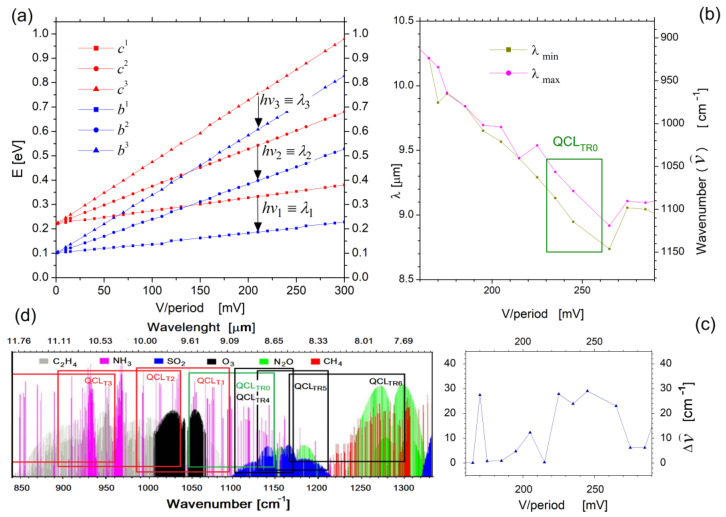
The QCL tuning simulations: (**a**) self-energies for three selected quantum states representing the *c*-miniband (in red) and the *b*-miniband (in blue) with marked electron transitions depending on the supply voltage; (**b**) the minimum and maximum values of the transition energies *hν*, converted to wavelengths *λ*, for the two observed minibands (*c* and *b*) with the effective tunable area QCL_TR0_; (**c**) illustration of wavenumber changes for the calculations shown in part (**b**); (**d**) absorption spectrum of chemical compounds plotted on the basis of HITRAN with marked QCL_TR0_ tuning area and QCL tuning areas after modifications of the active region described in the next chapter.

An earlier paper [14] has shown that the frequency range of the waves emitted by the laser can be changed by adjusting the QCL power conditions. In our case, the voltage applied to the superlattice structure was varied. Hence, the simulations of the laser in a wide range of applied voltages were performed to examine the possibilities of tuning QCL and possible matching of the emitted radiation to the absorption spectrum of the chemical substances to be detected. Selected results of such simulations are shown in Figure 4. Part (a) illustrates the following paths (in the energy domain) of three selected quantum states representing the *c* mini-band (in red) and the *b* mini-band (in blue), depending on the supply voltage of the structure. The calculations were performed under FMSL model for the superlattice five periods within the supply voltage range from 0 to 300 mV/period. The diagram shows the *h*ν transitions, analysed in terms of determining the emitted photons wavelength range. This range is represented by the values *λ_min_*= *h*c/Δ*E_max_* and *λ_max_*= *h*c/Δ*E_min_*, determined for each value of the applied voltage. The obtained results are plotted in part (b) of Figure 4, for the voltage ranging from 150 to 300 mV/period. In the same graph, the supply voltage range responsible for tuning the laser above the J_TH_ threshold, marked as QCL_TR0_ (tuning range 0), is highlighted. The covered voltage range was from 230 to 260 mV/period.

As shown in the QCL_TR0_ range, a relatively constant width of the emitted radiation is observed (represented by the parameter $\Delta \hat{\nu }$), which for the supply voltage 220 mV/period is comparable to the width of the radiation spectrum depicted in Figure 1b. The $\Delta \hat{\nu }$ values, however, tend to change with the supply voltage, which is most distinctly seen in Figure 4c. The exact values of this parameter, as well as changes in the wavelength *λ* converted from the energy *hν* for transitions between states *c* → *b,* are recorded in Table 2. By analyzing the results (FMSL), one can see that within QCL_TR0_ the laser is able to emit radiation in the wavelength range *λ* = 8.72 ÷ 9.52 μm (Δ*λ* = 0.8 μm), when calculated, wavenumber $\hat{\nu }$=1041 ÷ 1146 cm^−1^ (Δ$\hat{\nu }$ = 105 cm^−1^). This is illustrated in Figure 4d as a green rectangle plotted against the background of the absorption spectrum of chemical substances, which makes it possible to identify potentially detectable compounds, such as NH_3_, O_3_, N_2_O, or SO_2_.

For the supply voltages corresponding to the ends of the QCL_TR0_, structure simulations were performed. The calculations were made under the RSM model with the parameters given in Table 2. With analyzation of the obtained results, differences in relation to the FMSL results presented in the table have been found. They result from the approximate (disregarding electron scattering) and exact (AD + IR + ID + AP + OP + E-P + E-E considered) approach related to the FMSL and RSM models applied, respectively. Because these differences do not significantly affect the subject of the study (i.e., the QCL_TR_ tuning range), and because FMSL enables very fast calculations, it was decided to use this model as the leading one for further calculations. It is also worth noting that the QCL_TR0_ is close to the values obtained for measurements of similar structures [10]. This confirms the validity of further actions aimed at extending or changing the range of the emitted waves by modeling the dimensions of the laser structure, and its active area, in particular.

## 4. Modeling of QCL Active Region

**Figure 5:**
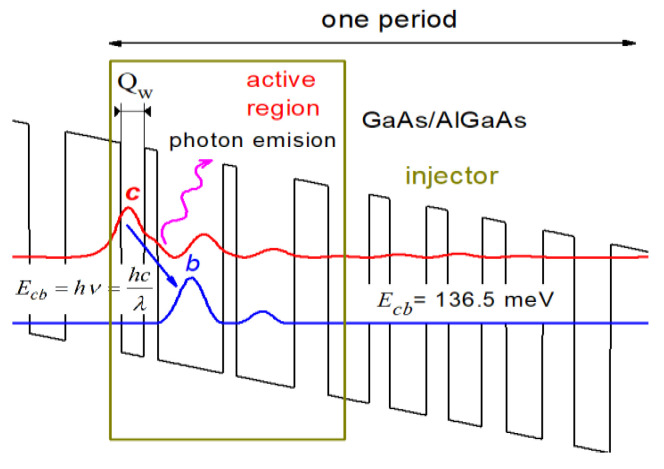
A scheme for modeling QCL active region.

Modeling of the active region of the selected QCL structure was carried out by adjusting the quantum well width for wells that contain the high quantum states (curve *c*) from which photon transitions to medium levels *b* are possible. This is schematically illustrated in Figure 5 where we graphically marked the Q_w_ parameter defining the concerned width of the well.

**Figure 6:**
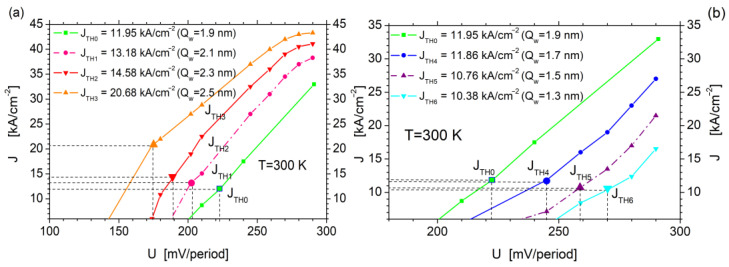
The fragments of current–voltage characteristics of the QCL with marked threshold currents for different widths of wells Q_w_ calculated using the RSM for the parameters listed in Table 1. Part (**a**) presents the results for increasing and (**b**) for decreasing the width of the well Q_w_.

Changing the spatial configuration of the QCL active region has a significant impact on its current–voltage characteristics. Thus, the maximum QCL tuning range calculated with the use of simplified FMSL had to be verified by determining the accurate value of its threshold current with an RSM for a given well width Q_w_. The simulation results are shown in Figure 6**,** where the fragmentary current–voltage characteristics of the tested structure for the selected well widths Q_w_ (ΔQ_w_ = ±0.2 ÷ 0.6 nm) are shown with threshold current values denoted. The values increase with the well width Q_w_ widened, while the related voltages for the structure supply tend to decrease to the value of 174 mV/period for Q_w_ = 2.5 nm, which corresponds to the threshold current J_TH_ = 20.68 kA/cm^−2^.

On the basis of the characteristics plotted in Figure 6a, the QCL_TR_ simulation results obtained under the FMSL approach were selected as presented in Table 3. The results show that increasing the well width Q_w_ reduced (as expected) the values of photon energy *hν*, which corresponds to longer wavelengths of the emitted radiation. For example, a change of ΔQ_w_= + 0.2 nm shifted the radiation spectrum to the range *λ* = 9.14 ÷ 10.1 μm, convertible to a wave number within the range $\hat{\nu }$ = 985 ÷ 1094 cm^−1^. Additionally, the QCL_T1_ tuning range extended in relation to QCL_TR0_, which was equal here to Δ*λ*_T1_ = 0.96 μm, corresponded to a wavenumber change within Δ$\hat{\nu }$ = 109 cm^−1^. Such a tendency has been observed for further widening of the well Q_w_, until the width of 2.5 nm for the range of wavenumber changes has reached Δ$\hat{\nu }$ = 161 cm^−1^. The range of the emitted radiation corresponded to *λ* = 10.42 ÷ 12.50 μm, and to a corresponding wave number within the range of $\hat{\nu }=799÷960$ cm^−1^.

The increased QCL tuning range resulting from widening the Q_w_ well width was also confirmed by calculations within the RSM approach, the results of which are represented by the values of the optical gain peaks shown in Table 3 as the parameter *hν*_mG_ converted also to the corresponding values of the radiation wavelength *λ*_mG_ and the wavenumber ${\hat{\nu }}_{mG}$. It can be seen here that the well widened to the width of 2.5 nm (which corresponds to approximately two monolayers) resulted in the extended laser tuning range from the initial QCL_TR0_ represented by the parameter Δ${\hat{\nu }}_{mG}$= 40 cm^−1^ to QCL_T3_, which corresponds to the value Δ${\hat{\nu }}_{mG}$ *=* 97 cm^−1^. This means that the emitted band had been extended nearly four times per wavenumber, which may open opportunities for detecting new chemical substances. The QCL_T3_ range, along with other parameters obtained by increasing the well width Q_w,_ are represented as red rectangles in the absorption spectrum plotted in Figure 4. It has been shown that within the basic tuning range (QCL_TR0_) the QCL module can be used to detect NH_3_ and SO_2_, whereas in the QCL_T2_ range the number of substances increases to three, specifically NH_3_, O_3_, and C_2_H_4_.

Narrowing the Q_w_ well width has led to increased *hν* energy, which in turn results in radiation of shorter wavelengths emitted by the laser. This has also affected the current–voltage characteristics of the QCL module and its threshold currents. The simulation results presented in Figure 6b, where the current–voltage characteristics of the QCL module for different (narrowed) Q_w_, together with the threshold currents and the corresponding supply voltages, are plotted, helped to confirm it. The presented graphs showed the QCL threshold current values to decrease slightly as Q_w_ narrows, and the corresponding structure supply voltages to be significantly increased in relation to the values presented on the base characteristic (in green).

Based on the characteristics plotted in Figure 6b, the QCL_TR_ simulation results obtained for the FMSL approach were selected as presented in Table 4. The results allow us to conclude that decreasing the well width Q_w_ quite expectedly increased the photon energy values *hν*, which corresponds to longer wavelengths of the emitted radiation. For example, a change of ΔQ_w_ = −0.2 nm shifts the radiation spectrum to the range *λ* = 8.54 ÷ 9.04 μm, which corresponds to the converted wave number within the range $\hat{\nu }$ = 1106 ÷ 1171 cm^−1^. The QCL tuning range here of the value of Δλ_T4_ = 0.5 μm (Δ$\hat{\nu }$=109 cm^−1^) is smaller than QCL_TR0_. Additionally, RSM simulations did not reveal any change within the emitted radiation energy in the considered tuning range (*hν_mG_* = 138 meV). Similar results were obtained for ΔQ_w_ = −0.4 nm, where *hν_mG_* =144 for U = 260 mV/period and U = 290 mV/period alike. A noticeable change, however, occurred for ΔQ_w_ = −0.6 nm, where the resulted change in *hν_mG_* corresponded to the value of 150 meV for the voltage of 290 mV/period. It corresponds to QCL_T6_ of a width of Δλ_T4_ = 0.91 μm (Δ$\hat{\nu }$ = 137 cm^−1^). 

All QCL tuning ranges obtained by narrowing the well width Q_w_ are plotted in Figure 4d as black rectangles. The results allowed us to assess the possibility of detecting chemical substances by modifying the laser structure. As shown, reducing the width of the well Q_w_ by 0.2 nm has not increased the laser tuning parameters. Similarly, a slight extension of the tuning range for QCL_T5_ has not introduced any new detection opportunities. Nevertheless, setting the width to Q_w_= 1.3 nm significantly widened the tuning range, and new chemicals, specifically N_2_O and CH_4,_ are likely to be detected with the QCL module modified accordingly.

## 5. Conclusions

The conducted research showed small changes introduced during building the QCL structure active region to be able to improve the tuning range significantly. Hence, new possibilities of detecting chemical substances by systems containing QCL modules are welcome. Such changes may be introduced by reducing or increasing just one or two monolayers in the quantum well, where photon transitions take place. Although the simulation results have not yet been confirmed by measurements, we believe this to be the right direction for changes in designing and producing modules to be applied in chemical substance detection systems.

## Figures and Tables

**Table 1:** The basic parameters of the QCL simulation for the results presented in Figure 2, Figure 3 and Figure 4.

GaAs/Al_045_Ga_0,55_As QCL	Well		Barrier
m*	0.067		0.10435
*E_g_* [eV]	0.84		1.84
*ε_r_*	12.85		13.8
Structure layers [nm] (barriers in bold)		**4.6**, 1.9, **1.1**, 5.4, **1.1**, 4.8, **2.8**, 3.4, **1.7**, 3.0, **1.8**, 2.8, **2.0**, 3.0, **2.6**, 3.0	
∆*E_C_* [eV]		0.39	
*n_dop_* [cm^−3^]		2.29 × 10^18^	
LO-phonon energy		0.036	
deformation potential [eV]		5.89	
Screening length *λ_Debye_* [nm]		32	
Number of periods QCL		30	
Temperature [K]		300	

**Table 2:** QCL tuning range calculated for the simulation results presented in Figure 2, Figure 3 and Figure 4.

Q_w_ [nm]	1.9					
U [mV/Period]	230		260		QCL_TR0_	
Model	FMSL	RSM	FMSL	RSM	FMSL	RSM
*hν*_min_ [meV]	129	126	138	129	9	3
*hν*_max_ [meV]	133	132	142	141	9	9
*hν*_mG_ [meV]		130		135		5
*λ*_min_ [μm]	9.31	9.38	8.72	8.78	0.59	0.6
*λ*_max_ [μm]	9.52	9.83	8.97	9.59	0.55	0.24
λ_mG_ [μm]		9.52		9.17		0.35
${\overset{⌢}{v}}_{\max}$ [cm^−1^]	1074	1066	1146	1139	72	73
${\overset{⌢}{v}}_{\min}$ [cm^−1^]	1041	1018	1116	1042	75	24
${\overset{⌢}{v}}_{mG}$ [cm^−1^]		1050		1090		40

**Table 3:** Modeling the QCL tuning range by extending the size of the Q_w_ well.

ΔQ_w_ [nm]	+0.2 (2.1)			+0.4 (2.3)			+0.6 (2.5)		
U [mV]	210	250	QCL_T1_	200	250	QCL_T2_	180	250	QCL_T3_
*hν_max_* [meV]	130	135	5	119	128	9	108	119	11
*hν_min_* [meV]	122	131	9	111	122	11	99	114	15
*hν_mG_* [meV]	120	126	6	114	120	6	102	114	12
λ*_min_* [μm]	9.52	9.14	0.38	10.40	9.67	0.73	11.46	10.42	1.04
λ*_max_* [μm]	10.1	9.46	0.64	11.14	10.15	0.99	12.50	10.89	1.61
λ*_mG_* [μm]	10.31	9.82	0.49	10.86	10.32	0.54	12.13	10.86	1.27
${\overset{⌢}{v}}_{\max}$ [cm^−1^]	1050	1094	44	902	1038	136	872	960	88
${\overset{⌢}{v}}_{\min}$ [cm^−1^]	985	1057	72	897	1007	110	799	918	119
${\overset{⌢}{v}}_{mG}$ [cm^−1^]	969	1017	48	920	969	49	824	921	97

**Table 4:** Modeling the QCL tuning range by narrowing the size of the Q_w_ well width.

ΔQ_w_ [nm]	−0.2 (1.7)			−0.4 (1.5)			−0.6 (1.3)		
U [mV]	250	290	QCL_T4_	260	290	QCL_T5_	270	290	QCL_T6_
*hν_max_* [meV]	142	145	3	145	150	5	154	161	7
*hν_min_* [meV]	137	141	4	140	148	8	144	156	12
*hν_mG_* [meV]	138	138	0	144	144	0	144	150	6
*λ_min_* [μm]	8.71	8.54	0.17	8.54	8.25	0.29	8.04	7.69	0.35
*λ_max_* [μm]	9.04	8.78	0.26	8.84	8.37	0.47	8.60	7.94	0.66
*λ_mG_* [μm]	8.97	8.97	0	8.60	8.60	0	8.60	8.25	0.35
${\overset{⌢}{v}}_{\max}$ [cm^−1^]	1147	1171	24	1171	1211	40	1243	1300	57
${\overset{⌢}{v}}_{\min}$ [cm^−1^]	1106	1139	33	1131	1195	64	1163	1260	97
${\overset{⌢}{v}}_{mG}$ [cm^−1^]	1115	1115	0	1163	1163	0	1163	1211	48

## Data Availability

Not applicable.
